# Evolution of tail fork depth in genus *Hirundo*

**DOI:** 10.1002/ece3.1949

**Published:** 2016-01-18

**Authors:** Masaru Hasegawa, Emi Arai, Nobuyuki Kutsukake

**Affiliations:** ^1^ Department of Evolutionary Studies of BiosystemsSokendai (The Graduate University for Advanced Studies)1560‐35 KamiyamaguchiHayama‐machiMiura‐gunKanagawa240‐0115Japan; ^2^ Division of Ecology and Evolutionary BiologyGraduate School of Life SciencesTohoku University6‐3 AobaAoba‐kuSendai, Miyagi980‐8578Japan

**Keywords:** Aerodynamics, flight habit, *Hirundo rustica*, migration, tail depth

## Abstract

A classic example of a sexually selected trait, the deep fork tail of the barn swallow *Hirundo rustica* is now claimed to have evolved and be maintained mainly via aerodynamic advantage rather than sexually selected advantage. However, this aerodynamic advantage hypothesis does not clarify which flight habits select for/against deep fork tails, causing diversity of tail fork depth in hirundines. Here, by focusing on the genus *Hirundo*, we investigated whether the large variation in tail fork depth could be explained by the differential flight habits. Using a phylogenetic comparative approach, we found that migrant species had deeper fork tails, but less colorful plumage, than the other species, indicating that migration favors a specific trait, deep fork tails. At the same time, tail fork depth but not plumage coloration decreased with increasing bill size – a proxy of prey size, suggesting that foraging on larger prey items favors shallower fork tails. Variation of tail fork depth in the genus *Hirundo* may be explained by differential flight habits, even without assuming sexual selection.

## Introduction

The deep fork tail of the barn swallow *Hirundo rustica* is a classic example of a sexually selected trait (Møller 1988; reviewed in Møller 1994a; Møller et al. 1998; Turner 2006; also see Scordato and Safran 2014 for a recent review). Males have deeper fork tails than females, and males with deeper fork tails have a mating advantage during social mate acquisition and extrapair mating (e.g., Møller 1988). However, since then, it has been proposed that aerodynamic advantage rather than sexually selected advantage is the main selection force driving deep fork tails in the barn swallow. This is because deeper fork tails provide better flight maneuverability than shallower fork tails when measured directly in the field or indirectly via a flight maze experiment (e.g., Norberg 1994; Evans 1998; Buchanan and Evans 2000; Park et al. 2000; Rowe et al. 2001).

One deficiency of the aerodynamic explanation is that it is unclear how the measured aerodynamic ability contributes to fitness in the wild (e.g., Barbosa 1999; Møller and Barbosa 2001; Cuervo and de Ayala 2005, 2014; but see Matyjasiak et al. 2013 for the link between aerodynamic ability and migration date in the barn swallow). Deep fork tails with streamer‐shaped outermost tail feathers (see Matyjasiak et al. 2004, 2009 for the relative role of length and shape) increase some aspects of aerodynamic ability (e.g., maneuverability) but impair others (e.g., flight velocity, acceleration), and thus, different depths of fork tail should be selected for in species with different ecologies and life histories (Park et al. 2000). Thus, it is necessary to investigate whether the tail fork depth (hereafter, “fork depth”) of a given species can, in fact, be predicted by its flight ecology when studying the importance of aerodynamics in the evolution of the fork tail. Previous studies have focused primarily on the deep fork tails of the barn swallow and tested the advantage of deep fork tails using species with shallow fork tails (e.g., the house martin *Delichon urbica*; Park et al. 2000) to study the initial evolution of fork tails. However, fork depths vary widely even within the genus *Hirundo* (*sensu* Sheldon and Winkler 1993). For example, males of *H. smithii* and *H. atrocaerulea* have fork tails of depth similar to those of *H. rustica* (>50 mm), others have shallow fork tails (e.g., *H. tahitica, H. nigrita*; <10 mm), and the remaining species have intermediate fork depths (e.g., *H. neoxena*,* H. dimidiata*; Table S1). The large variation in fork depth within genera provides a suitable opportunity to study the evolution of fork depth in relation to species‐specific flight habits, because members of this genus, like other hirundines, have a similar ecological background (e.g., feed on wing, monogamy; Turner and Rose 1994; Turner 2004) and morphologies well adapted to their strictly aerial insectivorous nature (e.g., long, pointed wing; Sheldon and Winkler 1993). Furthermore, all species in the genus *Hirundo* use a cup‐shaped nest for breeding (Turner and Rose 1994; Turner 2004; Sheldon et al. 2005). Nest shape (e.g., an enclosed nest) is a potentially severe constraint on the evolution of deep fork tails by damaging the fragile outermost tail feathers, which may explain why the house martin, a highly aerial insectivorous hirundine, lacks a fork tail (Park et al. 2000; Rowe et al. 2001; Matyjasiak et al. 2004). Use of the genus *Hirundo* allows investigation of flight habits, without being confounded by the nest shape. An alternative approach, the use of all hirundine species with nest shape as a covariate, cannot be applied because cup‐shaped nesters form a monophyletic group in the Hirundinidae (Sheldon et al. 2005), and thus, the effect of nest shape on fork depth cannot be statistically controlled. In addition, fork depth cannot be obtained in some species with shallow forks; in fact, the genus *Hirundo* represents the largest set of species with a complete dataset (Turner and Rose 1994).

Here, we investigated the evolution of fork depth in the genus *Hirundo* (14 described species: Dor et al. 2010) in relation to flight habits using a phylogenetic comparative approach. We focused on two aspects of flight habits: migration and foraging. Previous studies have shown that deeper fork tails enhance maneuverability but impair flight speed (Park et al. 2000; also see Matyjasiak et al. 2004). Recent studies have also shown that elongated tails mechanistically provide stability and lift during gliding in swallowtail butterflies (Park et al. 2010), which may also be applicable to swallows (see Norberg 1994 for lift generation in swallow tails in a steep rise; see Thomas 1996; Thomas and Taylor 2001 for the stability of bird tails in general). Swallows migrate during daytime and at low altitudes, where they will encounter air turbulence (Turner and Rose 1994; Turner 2006), and more maneuverable birds are expected to be better able to cope with turbulence (reviewed in Tobalske 2007). In fact, Matyjasiak et al. (2013) have recently been shown that male barn swallows with high aerodynamic ability (measured by principal component, characterized by high maneuverability, acceleration, and velocity) migrate to breeding sites faster than others, indicating that maneuverability might contribute to migratory habits. Thus, we predicted that migrant species have deeper fork tails than other species, because high maneuverability and stability would be advantageous during migration, particularly when they cannot avoid adverse weather by perching (e.g., when crossing wide geographic barriers such as oceans or deserts). On the other hand, rapid flight is needed to catch large, strong‐flying insects (e.g., Turner 1982; reviewed in Turner 2006). Although some authors have argued that maneuverability should also be important in capturing large prey (e.g., Buchanan and Evans 2001; also see Norberg 1994), the relationship between prey size and the turning ability is not straightforward compared to the relationship between prey size and the flight speed (Dudley 2002). Thus, we predict that species foraging on such nutrient‐rich, profitable food items should have shallower forks. This was also predicted by previous studies. Deeper‐forked swallows captured smaller prey items, perhaps by changing flight strategy (e.g., Møller 1989; Møller and de Lope 1994; Møller et al. 1995; Matyjasiak et al. 1999, 2000; Nudds and Spencer 2004), although the direct link between foraging ability and aerodynamic ability was not examined (see Matyjasiak et al. 2013). Because prey size itself is difficult to obtain from the literature (see “Materials and Methods” section), we used bill size as a proxy. To confirm that birds with larger bills collect larger prey (*sensu* Fitzpatrick 1985; also see Hespenheide 1971), we first investigated the relationship between bill size and prey size using available information and then tested the relationship between bill size and fork depth.

Fork depth is a sexually selected trait, at least in *H. rustica*, and sexual selection pressure might also explain the association between fork depth and flight habits if sexual selection pressure changes with flight habits. Thus, we also investigated chestnut plumage coloration, another sexually selected trait in the barn swallow (e.g., Ninni 2003; Safran and McGraw 2004; Hasegawa and Arai 2013a, 2013b; note that another characteristic of the genus *Hirundo*, blue‐black dorsal coloration, has little importance in sexual selection; Perrier et al. 2002; Galván and Møller 2009). By using two sexual traits (i.e., fork depth and plumage coloration), we can distinguish trait‐specific selection from general selection on sexual traits. If migration and small prey size somehow favor sexual selection in general, we predict that fork depth and chestnut coloration will exhibit a similar pattern; however, aerodynamic predictions can be applied solely to fork depth.

## Materials and Methods

### Data collection

Information regarding fork depth (i.e., the difference between the tips of the inner and outer tail feathers; Turner 2006, p. 24), migratory habits, and bill size was obtained from Turner and Rose (1994). Migratory habits were divided into two categories: migrants and others. We regarded species as migrant when they had breeding sites separated from wintering sites (i.e*.,* when they were a summer visitor in a portion of their distribution range, which is shown in yellow in plates in Turner and Rose 1994; also see Turner 2004). We used mean fork depth and bill length as representatives of the fork depth and bill size of the species, respectively. Although other bill morphologies (e.g., bill depth) cannot be obtained from the literature, bill length is in fact predicted to vary functionally with prey size in aerial insectivorous birds (Fitzpatrick 1985, p. 452). When information about multiple subspecies was available, we used data on the nominate subspecies. When sex‐specific values could be obtained (*n* = 7; note that the lack of a sex‐specific value does not always mean a lack of sexual dimorphism, as in *Hirundo neoxena*; Turner and Rose 1994, p. 177), we used those of males, because the tail fork depth in females might be suboptimal due to intersexual genetic correlations with optimal male homologous traits (see Møller 1993; Cuervo et al. 1996; Evans 1999). Because of the large variation of measurements within species, trait sizes were rounded down to the nearest integer so as not to overfit the model. Similarly, we also used the mean wing length of the species as a representative of species body size to account for the potential confounding effect of body size (Turner and Rose 1994; typo was corrected based on a personal communication from Angela Turner, i.e., the wing lengths of *H. angolensis* and *H. neoxena* are not 199 mm and 122 mm, but 119 mm and 112 mm, respectively). We also gathered information about prey items from Turner and Rose (1994). Species were classified into birds foraging on large prey items and others: large prey items included “dragonflies,” “large‐sized flies,” “vespiform,” “tabanids,” “carabids,” “large flies,” and “wasps”; these were distinguished from other prey items (e.g., ants, termites, midges, mayflies). For *H. dimidiata* and *H. leucosoma*, descriptions have noted that “details of their diet are not known” and “the diet…(omitted) is not known in details,” respectively; thus, these were excluded from analysis of the relationship between bill size and prey size. We summarize this information in Table S1. Because bill size was not significantly related to migration (Difference in bill length [migratory species – other species] ± SE = 0.97 ± 0.56, 95% CI = –0.29, 2.22; this was also the case even after statistically controlling for body size measured as log(wing length): data not shown), a potential confounding effect of Allen's rule, that is, relatively shorter appendages in colder environment (Nudds and Oswald 2007), would be negligible.

Chestnut plumage coloration, possibly pheomelanin‐based coloration (McGraw et al. 2004), was scored following the method described in Soma and Garamszegi (2015) using plates in Turner and Rose (1994). In short, we considered chestnut plumage coloration as an ornamental trait (e.g., Safran and McGraw 2004) and measured the coverage of the ornamental trait on the whole body surface. Body surface was divided into 21 plumage regions (i.e., belly, breast, chin, throat, cheek, crissum, crown, flank, forehead, greater primary coverts, greater secondary coverts, lore, lesser wing coverts, mantle, median wing coverts, nape, primaries, rectrices, rump, secondaries, and upper tail coverts; see figure 2.10 in Andersson and Prager 2006). We scored each region as 0 when not colored, 0.5 when partially colored, and 1 when totally colored and summed these scores across bodies. In the current case, chestnut coloration was found on the belly, frank, breast, chin, throat, cheek, crissum, crown, and forehead. Minimum vales were zero, which were assigned to *H. nigrita* and other black‐and‐white swallows, which have no chestnut plumage patch; the maximum value was 6.5, for *H. negrorufa*, which has chestnut plumage coloration across its ventral surface (Table S1).

### Phylogenetic comparative analysis

All analyses were carried out in R 3.0.2 (R Development Core Team 2013) using the packages “ape” (Paradis et al. 2004) and “caper” (Orme 2012). To account for phylogeny, we used the phylogenetic general least square model (PGLS; function pgls in the caper package). We used 9999 alternative trees of the genus *Hirundo* from birdtree.org. To account for phylogenetic uncertainty (see Fig. S1 for a consensus tree), we fitted models to each tree and applied multimodel inference (Garamszegi and Mundry 2014), following the example codes at http://www.mpcm-evolution.org/practice/online-practical-material-chapter-12/chapter-12-2-dealing-phylogenetic-uncertainty-inference-across-models-considering-different-phylogenetic-hypotheses. Because estimates of *λ* values, which are sensitive to sample size, are a poor measure of phylogenetic signal in a small sample (i.e., numbers of species; Symonds and Blomberg 2014, p. 119), we assumed a strong phylogenetic signal, as is expected for morphological traits (i.e., assuming a Brownian motion model with *λ *= 1; Symonds and Blomberg 2014, p. 117). However, when *λ* values were changed to 0.8, 0.6, 0.4, 0.2, and 0.000001 (i.e., minimum *λ* value in function pgls), we obtained qualitatively similar results (i.e*.,* significant and nonsignificant predictors remained significant and nonsignificant, respectively; see Figs. S2, S3). Thus, our models were robust for the uncertainty of *λ* values. We derived model‐averaged mean coefficients, SEs, and 95% confidence intervals (CIs) via model averaging. When calculating 95% CIs, we used *t*‐values based on appropriate degrees of freedom (i.e., in all cases, df = 10 and, thus, *t *=* *2.228 were used), because of the small sample sizes (*n *<* *30; Symonds and Blomberg 2014). We also presented model‐averaged pseudo‐*R* ^2^_adj_ values. To account for differential body sizes across species, we included log(wing length) together with migration and bill size when studying log(fork depth) and plumage coloration. Although the sample size of the current study was relatively small (i.e., 14 species) compared with the number of predictor variables, we gave priority to the correct model, as suggested by Mundry (2014). When we tested for multicollinearity among variables using the variance inflation factor (VIF; a VIF of **>**2.5 would be problematic), we found very low VIF values (max = 2.2), indicating that multicollinearity may have few effects on the predictors.

## Results

Species foraging on large prey items had larger bills than did other species (*n *=* *12, Difference in bill length [species foraging on large prey items – other species] ± SE = 1.54 ± 0.56, 95% CI = 0.30, 2.78). This relationship confirmed that bill size reflects prey size, at least in part. General body size did not confound the relationship, because there was no significant difference in log(wing length) between the two groups (*n* = 12, Difference ± SE = 0.06 ± 0.04, 95% CI = −0.04, 0.16; note that we obtained qualitatively similar results without log transformation).

**Figure 1:**
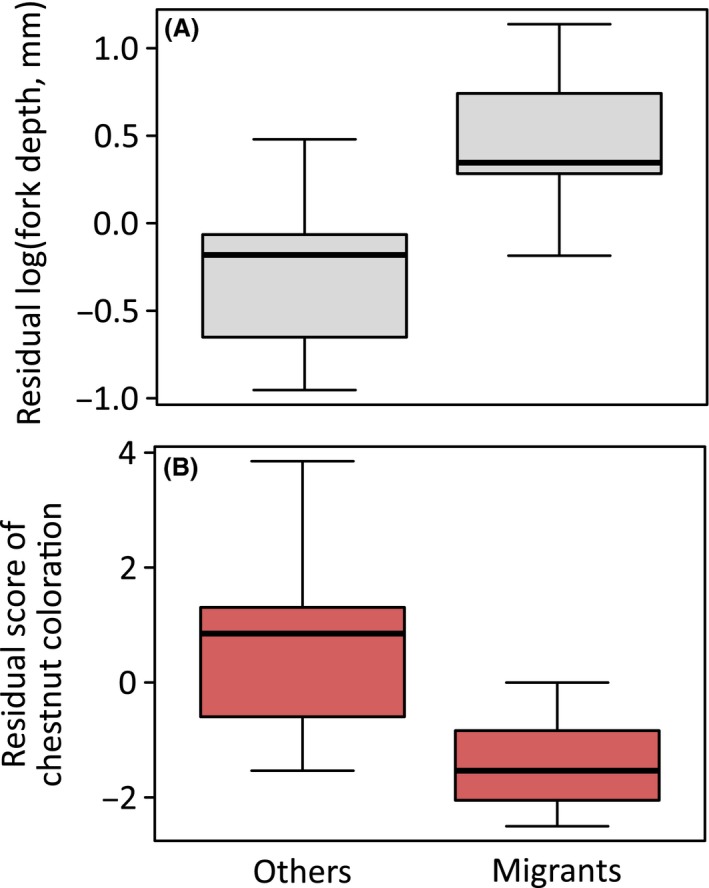
Boxplots showing (A) residual log(fork depth) and (B) residual chestnut coloration score by each species category after controlling for covariates (see Table 1). The bars, boxes, and whiskers in each boxplot indicate the medians, the first and third quartiles of data, and the lowest to the highest data points, respectively.

**Figure 2:**
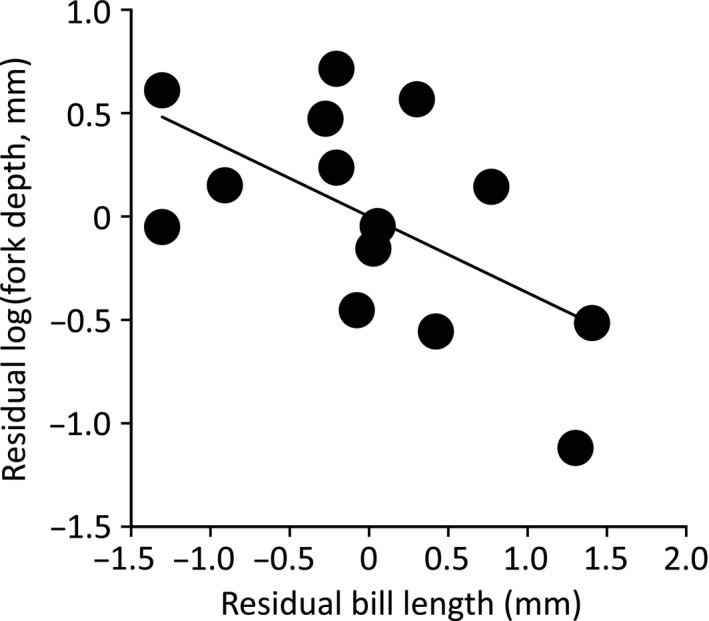
Residual log(fork depth) in relation to bill length – a proxy of prey size – after controlling for covariates (see Table 1). The line is a simple regression between the residuals.

After controlling for body size using log(wing length), migration and bill size significantly explained the variation in log(fork depth): migrant species had deeper fork tails than did other species (Table 1; Fig. 1A). In fact, the three species with the deepest forked tails, *H. rustica*,* H. smithii*, and *H. atrocaerulea* (all > 50 mm), were migratory species, whereas the two species with the shallowest forked tails, *H. neoxena* and *H. dimidiata* (both < 10 mm), were included as the other group (Table S1). Moreover, fork depth increased with decreasing bill size, which is a proxy of prey size (Fig. 2).

**Table 1:** Multivariable phylogenetic generalized least square (PGLS) model for log(fork depth) and chestnut plumage coloration (*n* = 14 each)

Predictor	Coefficient ± SE	95% CI
Response: log(fork depth)		
**Migrants or not**	**1.48** ± **0.32**	**0.78 to 2.19**
**Bill length**	−**0.39** ± **0.15**	−**0.72 to** −**0.06**
log(wing length)	2.54 ± 2.42	−2.85 to 7.94
Response: plumage coloration		
**Migrants or not**	−**2.75** ± **1.17**	−**5.37 to** −**0.14**
Bill length	−0.62 ± 0.56	−1.87 to 0.62
**log(wing length)**	**25.40** ± **8.96**	**5.43 to 45.36**

Model‐averaged coefficient, SE, and 95% confidence intervals (CI) are shown (model‐averaged pseudo‐*R* ^2^_adj_ values for fork depth and plumage coloration were 0.70 and 0.36, respectively). Significant test results (i.e., 95% CI does not contain zero) are indicated in bold.

In contrast, when controlling for the significant positive link to body size measured as log(wing length), chestnut plumage coloration was explained by migration but not by bill size (Table 1). Migrant species had a lower chestnut coloration score than the other species, indicating that migrant species were less ornamented (Fig. 1B). This result contrasts with those for fork depth.

## Discussion

The current finding is consistent with the predictions that migratory species have deeper fork tails than others and that fork depth decreased with increasing bill size, a correlate of prey size. To our knowledge, this is the first phylogenetic evidence that variation of fork depth could be explained by differential flight habits, reinforcing the importance of flight habits in the evolution of fork depth in the genus *Hirundo*. These results are also consistent with within‐species patterns, as (more) migrant populations possess deeper forks in *H. rustica*,* H. smithii*, and *H. albiguralis* (e.g., Turner and Rose 1994; Møller 1995a; Turner 2004; Dor et al. 2012), whereas individuals of *H. rustica* and other hirundines with shallower fork tails captured larger prey (e.g., Møller 1989; Møller and de Lope 1994; Møller et al. 1995; Matyjasiak et al. 1999, 2000). Clearly, the current finding is inconsistent with the hypothesis that deep fork tails can function to capture large prey items (Buchanan and Evans 2001), although they might still be effective for capture of many (but small) prey items.

Of the two potential sexual traits (see “Introduction”), fork tail depth, but not chestnut plumage coloration, showed the predicted relationships (Table 1); thus, it is unlikely that migrant species have high total selection for sexual traits in general due to the intense sexual selection pressure. Rather, expression of each trait would depend on trait‐specific costs and benefits, which deserve further attention here. Concerning chestnut plumage, why migratory birds are less colorful is unknown. One possible mechanism is differential oxidative stress. Migration accompanies more oxidative stress and thus should not develop chestnut, pheomelanin‐predominant coloration that consumes an important antioxidant (glutathione) during pheomelanogenesis (e.g., Galván et al. 2010; Roulin et al. 2011; also see Norris et al. 2009 for a possible tradeoff between migration activity and pheomelanogenesis in the barn swallow). It remains to be clarified whether this explanation or some other explanations can be applicable to the genus *Hirundo*.

Concerning fork depth, a classic explanation of the geographic variation is the balance between natural selection and sexual selection on fork depth. High‐latitude populations should have deeper fork tails due to intense sexual selection there (Aparicio et al. 2012), or migratory birds that breed in high‐latitude zones might experience relaxed selection pressure against deep fork tails, which are costly for foraging large, strong‐flying insects (because prey items should be slow in low‐temperature zones, i.e., high latitudes, due to the temperature‐dependent activity; Møller 1995a). Both of these explanations assume intense sexual selection for deep fork tails (also see Møller et al. 2006). However, sexual selection pressure might be weaker than thought previously, even in the model species, the barn swallow *H. rustica* (e.g., ca. 10 mm of the outermost tail feathers might be responsible for sexual selection; Buchanan and Evans 2000; note that this is 16% of fork depth; Table S1), and all other *Hirundo* species, with the exception of *H. atrocaerulea,* have shallower fork tails than *H. rustica* (see Table S1), implying a much smaller contribution of sexual selection to fork depth in the genus *Hirundo* in general. In fact, even when the seemingly sexually selected portion of fork depth (i.e., 16%; see above) was subtracted from the total fork depth of each migratory species, the effect of migration on fork depth remained significant (Fig. S4), indicating that migrants have fork tails deeper than predicted from sexual selection alone. The naturally selected portion of fork depth should be increased in migratory species, perhaps via aerodynamic advantage.

This simple explanation, the aerodynamic advantage of a deep fork tail at migration, can also explain why individuals with deeper fork tails have several advantages for migration (e.g., the good condition, early migration, and survival advantages of individuals with deep fork tails in the barn swallow; e.g., Møller 1994a, 1994b; Matyjasiak 2013; also see Brown and Brown 1999). Researchers should not assume that migration has detrimental effects on deep fork tails but should test the direction of selection at migration. Such studies are limited even in the model species, *H. rustica* (see Saino et al. 1997 for an exception; although the cost measurement, hematocrit value, might be problematic; Cuervo and de Ayala 2005, 2014); thus, viability selection on deep fork tails during migration should be clarified using appropriate measures of fitness, ideally while controlling for foraging cost.

The relationship between flight habits and fork depth might also explain the sex difference in fork depth. It is well known that male barn swallows migrate earlier (i.e*.,* in more severe weather conditions) than females, whereas females invest more into feeding young than males (Turner 2006), indicating that the relative importance of migration/foraging is higher in males than females (also see Kokko et al. 2006). This sex difference in flight habits might cause sex‐specific selection and thus be responsible for sexual dimorphism in fork depth and related flight characteristics as well (e.g., aspect ratio; Møller 1995b; Matyjasiak et al. 2013; also see Evans and Thomas 1997). The resultant morphological difference might be amplified via sexual selection on the trait itself, because the offspring of males with higher survivorship may inherit this trait and thus females should choose such males as a mate, yielding a runaway process (Bro‐Jørgensen et al. 2007). In summary, differential flight habits can overcome the deficiencies of previous aerodynamic explanations of deep fork tail: (1) functional importance (i.e., at migration but not at foraging) and (2) causation of sexual dimorphism (i.e., via a sex difference in viability selection at migration).

## Conflict of Interest

None declared.

## Supporting information


**Figure S1**. Consensus phylogenetic tree of the genus *Hirundo* derived from the function “consensus” in the R package “ape” using 9999 trees obtained from birdtree.org (see “Materials and Methods” for detailed information).

**Figure S2**. Univariable phylogenetic generalized least square (PGLS) model for bill size with varying *λ* values (*n *= 12).

**Figure S3**. Multivariable phylogenetic generalized least square (PGLS) model for log(fork depth) and pheomelanic plumage coloration with varying *λ* values (*n *= 14 each; left column; log(fork depth); right column: plumage coloration).

**Figure S4**. Multivariable phylogenetic generalized least square (PGLS) model for log(adjusted fork depth) with varying *λ* values (*n *= 14 each).

**Table S1**. Dataset of the current study using all 14 species of the genus *Hirundo* from Turner and Rose (1994).
