# Supplementary material for: Evolution of tail fork depth in genus Hirundo
Source: Ecol Evol. 2016 Jan 18;6(3):851–8. doi: 10.1002/ece3.1949 (PMC4739571; doi:10.1002/ece3.1949)

Figure S1

Consensus phylogenetic tree of the genus *Hirundo* derived from the function "consensus" in the R package "ape" using 9999 trees obtained from birdtree.org (see Methods for detailed information).

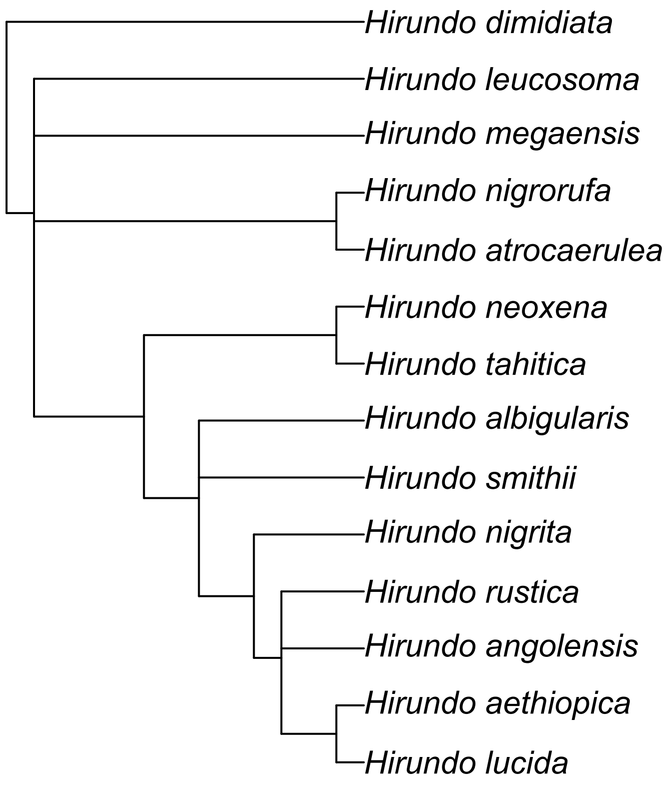

Supplement: Supplementary file 1 — Figure S1. Consensus phylogenetic tree of the genus Hirundo derived from the function “consensus” in the R package “ape” using 9999 trees obtained from birdtree.org (see “Materials and Methods” for detailed information). [file ECE3-6-851-s001.pdf]
