# Supplementary material for: Evolution of tail fork depth in genus Hirundo
Source: Ecol Evol. 2016 Jan 18;6(3):851–8. doi: 10.1002/ece3.1949 (PMC4739571; doi:10.1002/ece3.1949)

Figure S4

Multivariable phylogenetic generalized least square (PGLS) model for log(adjusted fork depth) with varying λ values (n = 14 each). Adjusted fork depth was computed by subtracting the seemingly sexually selected portion of fork depth (16%; see text) from the total fork depth of each migratory species. Model-averaged coefficient and 95% confidence intervals (CI) of migration, bill length, and log(wing length) in relation to λ values (0.000001, 0.2, 0.4, 0.6, 0.8, 1.0) are shown. Significant test results (i.e. 95% CI does not contain zero) are indicated by asterisks. Detailed information is provided in the text.

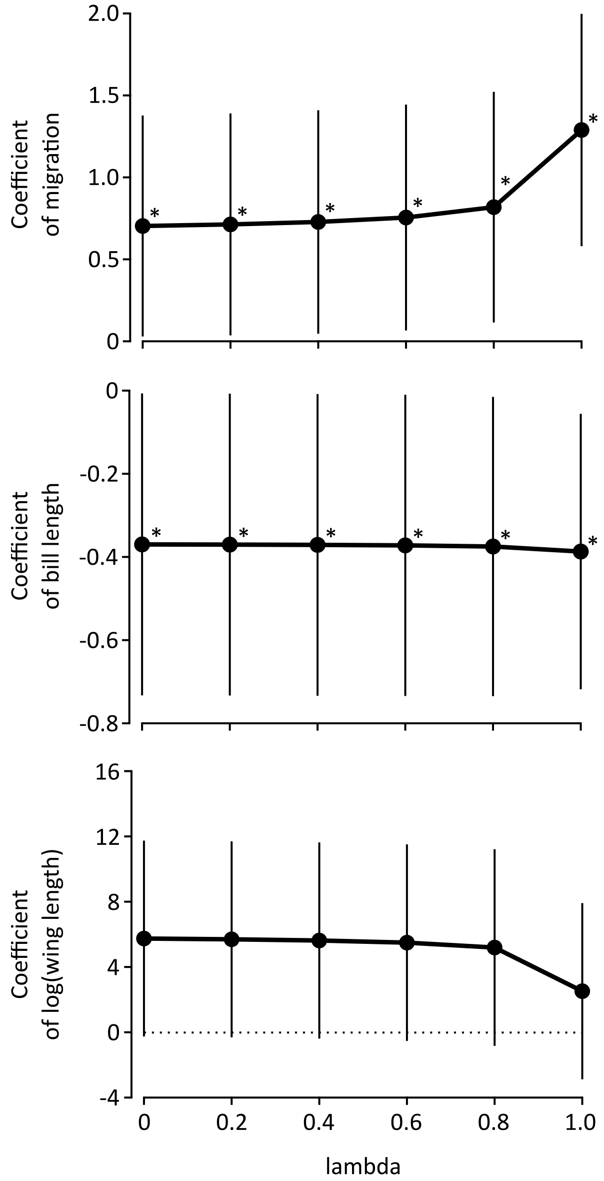

Supplement: Supplementary file 4 — Figure S4. Multivariable phylogenetic generalized least square (PGLS) model for log(adjusted fork depth) with varying λ values (n = 14 each). [file ECE3-6-851-s004.pdf]
