# Supplementary material for: Evolution of tail fork depth in genus Hirundo
Source: Ecol Evol. 2016 Jan 18;6(3):851–8. doi: 10.1002/ece3.1949 (PMC4739571; doi:10.1002/ece3.1949)
Supplement: Supplementary file 5 — Table S1. Dataset of the current study using all 14 species of the genus Hirundo from Turner and Rose (1994). [file ECE3-6-851-s005.docx]

**Table S1**

Dataset of the current study using all 14 species of the genus *Hirundo* from Turner and Rose (1994). See Methods section for detailed information (typo was corrected based on a personal communication from Angela Turner).

| Species Wing length Fork depth Migrants? Bill length Coloration  (mm) (mm) (yes = 1) (mm) (score) |
| --- |
| *H. aethiopica* 106 27 0 10 1.0  *H. albigularis* 128 32 1 12 1.0  *H. angolensis* 119 16 0 11 3.5  *H. atrocaerulea* 113 92 1 10 0.0  *H. dimidiata* 102 24 1 9 0.0  *H. leucosoma* 99 13 0 9 0.0  *H. lucida* 111 21 0 10 3.5  *H. megaensis* 102 23 0 9 0.0  *H. neoxena* 112 31 0 9 4.0  *H. nigrita* 106 5 0 11 0.0  *H. nigrorufa* 112 16 0 9 6.5  *H. rustica* 124 61 1 12 2.5  *H. smithii* 110 50 1 9 2.0  *H. tahitica* 105 9 0 11 4.0 |

Among these species, *H. albigularis, H. neoxena, H. nigrita, H. rustica,* and *H. tahitica* were recorded to capture large-sized prey items, and their bill lengths were compared with those of the remaining species, excluding *H. dimidiata* and *H. leucosoma*, for which there was insufficient data (see Turner and Rose 1994; see also text).
